# MicroRNA Shuttle from Cell-To-Cell by Exosomes and Its Impact in Cancer

**DOI:** 10.3390/ncrna5010028

**Published:** 2019-03-21

**Authors:** Heidi Schwarzenbach, Peter B. Gahan

**Affiliations:** 1 Department of Tumor Biology, University Medical Center Hamburg-Eppendorf, 20246 Hamburg, Germany; 2 Fondazione “Enrico Puccinelli” Onlus, 06126 Perugia, Italy; peterbgahan@gmail.com

**Keywords:** microRNA biogenesis, microRNA functions, microRNA pathways, exosome biogenesis, exosome functions, exosomal signaling, breast cancer, epithelial, ovarian cancer, prostate cancer

## Abstract

The identification of exosomes, their link to multivesicular bodies and their potential role as a messenger vehicle between cancer and healthy cells opens up a new approach to the study of intercellular signaling. Furthermore, the fact that their main cargo is likely to be microRNAs (miRNAs) provides the possibility of the transfer of such molecules to control activities in the recipient cells. This review concerns a brief overview of the biogenesis of both exosomes and miRNAs together with the movement of such structures between cells. The possible roles of miRNAs in the development and progression of breast, ovarian and prostate cancers are discussed.

## 1. Introduction

Originally, membrane vesicles were considered as cell debris and signs of cell death; they were known as micro-particles when present in blood and as prostasomes when in seminal fluid [1]. However, the understanding of these vesicles was changed by Harding et al. (1983) with their study on transferrin recycling [2]. In this and subsequent studies [2,3], the authors demonstrated the uptake of transferrin by endocytosis, but with the endosomes not being merged with primary lysosomes and instead passing either directly to multi-vesicular bodies (MVBs) or being recycled to the cell membrane as apo. Thus, transferrin receptors (Tf-R) appeared to be either recycled or selectively released from reticulocytes. It was proposed that this occurred by (i) uptake of the diferric transferrin via endocytosis followed by the production of a pleiomorphic class of small vesicles and tubules with either (ii) removal of iron from transferrin for transfer to the cytoplasm or (iii) the apo-transferrin-receptor complex recycling to the plasma membrane where apo-transferrin separates from the receptor or (iv) those Tf-Rs due to leave the cell segregate in inclusion vesicles of MVBs for release by MVB exocytosis.

Interestingly, these MVBs showed no reaction for selected acid hydrolases as would be expected of the lysosomal system components. In early studies on the lysosomal system, MVBs have been found to be present in a range of normal and pathological animal tissues, yet they were frequently demonstrated to contain acid hydrolases [4,5]. The MVBs were thought to contain the final debris from the breakdown of certain structures prior to forming the residual body that would eliminate its contents by exocytosis. Thus, the study of Harding et al. (1983) was one of the first to offer a functional role for MBVs from which acid hydrolases appeared to be absent [2].

The term exosome, applied to such vesicles released from the MBVs and isolated by centrifugation at 100,000× *g* for 90 min, was suggested by Johnstone et al. (1987) working with sheep reticulocytes. They proposed that the exosomes offered a mechanism for the removal of specific membrane functions that are seen to reduce during reticulocyte maturation [6]. Initially, exosomes were considered to have RNase associated with the membrane in order to destroy the apparently unwanted RNA that was present therein [7]. 

In contrast, exosomes are spherical, about 30–150 nm in diameter and were also found to contain a range of molecules including DNA, RNA, protein and lipids. Exosomes also carry intraluminal and transmembrane proteins e.g., heat shock proteins (HSP70, HSP90), integrins, and tetraspanin proteins (CD9, CD63, CD81, CD82), as well as those proteins involved in both membrane transport and fusion (e.g., Rab GTPases, annexins, flotillins) and MVB biogenesis (e.g., Alix and tumor susceptibility gene 101 or Tsg101). In addition, ceramides, and cholesterol have been shown to be present [8,9].

Exosomal DNA, both genomic (100 b–17 kb) and mitochondrial, has been found in both normal and pathological environments [10,11,12,13,14]. Under experimental conditions, DNA-containing exosomes have been linked to the initiation of both glioma and colorectal cancer [10,14]. 

Importantly, exosomes have been found to contain a wide range of RNAs including mRNA, miRNA, rRNA, tRNA, lncRNA, piRNA, snRNA and small nucleolar RNA. Using deep-sequencing, Huang et al. (2013) were able to characterize human plasma-derived exosomal RNAs, the dominant fraction being mRNA at 42.32%. The other fractions were rRNA (9.16% of all mappable counts), lncRNA (3.36%), piRNA (1.31%), tRNA (1.24%), snRNA (0.18%) and small nucleolar RNA (0.01%). The five most common of the 593 miRNAs detected were miR-99a-5p, miR-128, miR-124-3p, miR-22-3p, and miR-99b-5p, collectively accounting for 48.99% of all mappable miRNA sequences [15]. Many thousands of miRNAs are present in a wide range of organisms appearing to be spread throughout the genome [16,17]. Rodrigues et al. [16] and Griffiths-Jones et al. [17] have listed 2588 annotated miRNAs in the human genome. Since each miRNA is thought to regulate the expression of hundreds of target genes, the miRNA pathway as a whole forms a critical way of controlling gene expression. Subsequent experiments have indicated roles for miRNAs in both healthy individuals and cancer patients including the possibility of exploiting some circulating miRNAs in cancer screening. 

The present review will consider the biogenesis of both exosomes and miRNAs and the role of miRNAs in breast, ovarian and prostate cancers.

## 2. Biogenesis and Function of miRNAs

### 2.1. miRNA Biogenesis Pathway

All miRNA host genes are transcribed by RNA polymerase II to form primary miRNA (pri-miRNA) transcripts [18,19]. These undergo typical splicing, capping, and polyadenylating similarly to protein-coding mRNAs thus leading to the formation of the active, 21–23 nucleotide, mature miRNAs [20]. The mature miRNA is integrated into the RNA-induced silencing complex (RISC) that targets mRNAs, resulting in translational repression and mRNA degradation

The main feature of all primary miRNA transcripts is the stem-loop precursor RNA (pre-RNA) with either one or, sometimes, both strands of the stem forming the mature miRNA. This is achieved by cleavage by the enzyme Drosha [21,22]. A number of steps are needed for the formation of mature miRNA and integration with the RISC complex. Further cleavage of the hairpin-like structure by the enzyme Dicer results in the mature miRNA [23,24]. RISC is an RNA-protein complex containing Argonaute protein that involves single-stranded RNA acting as a template for RISC in its recognition of the RNA to be broken down. Once found, RISC activates and cleaves the mRNA. 

There are a number of alternative routes to the production of miRNAs including one in which mirtrons bypass the Drosha processing step, the precursor being produced by a splicing reaction. The subsequent steps with RISC are similar.

### 2.2. Drosha and Dicer

The primary miRNA (pri-miRNA) requires two endonucleases to act on it prior to its maturation as active miRNA, namely, Drosha and Dicer. The first step is nuclear based and involves the enzyme Drosha [25] occurring either during or subsequent to pri-miRNA transcription. In this process, Drosha associates with the RNA binding protein DGCR8 [23,26,27] resulting in the stem-loop precursor release from the flanking pri-miRNA transcript sequences. 

Subsequent to its export from the nucleus in a Ran-GTPase dependent fashion by Exportin5 [28], Dicer, in association with the RNA binding protein TRBP, cleaves the precursor loop region so releasing mature miRNA [29]. This reaction yields a duplex RNA of 21–23 nucleotides long, depending on the miRNA. One of the strands is integrated into RISC as a mature miRNA whilst the other strand, the star strand, is usually degraded. However, sometimes, both strands can be integrated into RISC. It appears that a small fraction of star strands can be loaded for almost all miRNA families [30]. 

miRNAs can use different strands dependent upon the cell type involved or the biological state [31]. Hence, 5p/3p are the terms used to name the strands depending on the strand being from the 5′ end of the stem-loop or the 3′ end, respectively.

### 2.3. RISC Loading and Targeting

The miRNA is incorporated into the protein complex RISC the exact composition of which is unknown. However, it does contain the Argonaute protein, four family members of which are present in humans (Argonautes—Ago1-4) [32]. At this stage, the star strand—usually 3′—is cut by Ago2 and further degraded by the nuclease complex C3PO [33,34]. Argonaute links directly with mature miRNA and identifies the target mRNAs with miRNA complementarity. Targeting is especially reliant on nucleotides 2–7 of the miRNA, termed the “seed” region [35,36].

### 2.4. Alternative Pathways

Most known miRNAs are produced by the canonical pathway. However, there are a number of alternative biogenesis pathways, of which the mirtron class of miRNAs is the most frequently observed [37,38]. Such miRNAs e.g., miRNA-451, are present in introns at the exon junction site, and exclude the Drosha stage. The precursors result from splicing cleavage events prior to the stem-loops being cut by Dicer and integrated into RISC. miRNA biogenesis independent of dicer has also been described [39,40]. Interestingly, other small RNAs e.g., tRNA, although not miRNAs, are produced by Drosha and/or Dicer activity and bound to RISC [41]. 

Some miRNAs appear to function independently of the RISC complex. A comparison of the total miRNA population in a cell with the RISC protein copy number implied that a large fraction of miRNAs is not associated with RISC [42]. Although the functional roles of RISC-free miRNAs are unknown, miR-328 has been reported to be complexed with the RNA binding protein hnRNP E2 [43]. This miRNA appears to block the linkage of the RNP with its target mRNA C/EBPα so enhancing C/EBP protein translation. However, such non-canonical pathways seem to be unusual since e.g., miR-328 mainly acts as a canonical miRNA within the RISC complex [44,45].

### 2.5. miRNA Target Identification

There are three general approaches to identifying miRNA targets namely, (i) bioinformatic target prediction, (ii) biochemical isolation of miRNA/mRNA complexes and (iii) transcriptomic/proteomic analysis [30]. Such target sets for miRNAs are needed so as to (a) understand their biological roles, (b) identify tumor types in both clinical and liquid biopsies and (c) develop possible miRNA therapeutics. 

### 2.6. Biological Roles of miRNAs

Mouse models deficient for Dicer and DGCR8 were first used to demonstrate that the loss of one or other stage of the biogenesis of miRNAs resulted in embryonic lethality [46,47]. Moreover, the lack of either gene from a specific tissue resulted in it being developmentally defective since i.e., a specific miRNA function is needed in most tissue types [48]. Generally, single miRNAs alone do not specify individual tissue development. Thus, mice lacking cardiac-specific miRNA miR-208 still develop a heart [49,50] but will show a defective stress response and cardiac hypertrophy. Hence, miRNAs are involved in the maintenance of the differentiated state of tissues. Indeed, often there is a reduction in tissue-restricted miRNAs for a variety of diseases, including cancer [30].

### 2.7. miRNAs in Cancer

miRNA expression changes in cancer have been shown by gene-profiling analyses, both in identifying cancer and its progression. miRNA expression alterations can be related to many types of cancer with early miRNA studies showing them to be either up-regulated, e.g., miR-21 and the miR-17–92 cluster, or down-regulated, e.g., miRNA families including the let-7 and miR-34 families [51]. Tissue-specific miRNAs have been often shown to be reduced in cancer, e.g., liver-specific miR-122 in hepatocellular carcinoma [52,53]. Some miRNAs may either promote or prevent cancer development and progression as has been determined by functional studies, e.g., the increase in the miR-17-92 cluster in many cancer types [54]. The involvement of such miRNAs in cancer pathogenesis promotes the concept of their development as therapeutic targets [55]. With a range of miRNA families altered in cancers, it will be of interest to develop miRNA signatures for both clinical diagnoses from tissue samples and liquid biopsy.

### 2.8. miRNAs in Liquid Biopsy

In spite of the presence of RNase in blood, the levels of which can rise in the presence of cancer [56], miRNAs were seen to survive due to their presence in exosomes. Thus, they provide a useful set of possible markers for liquid biopsy analysis—for the monitoring of the presence, treatment and reappearance of a particular cancer. 

## 3. Biogenesis and Functions of Exosomes

Exosomes are the vesicles exploited by both healthy and pathological cells to transfer various RNAs, and miRNAs in particular, to other recipient cells so acting as a messenger vehicle between cells. An exosome appears to contain more than one miRNA and it is not clear either how they are selected for uploading or if there are miRNAs that co-operate simply/synergistically or in a mutually inhibitory fashion.

The manner in which the exosomal vesicles are formed, the mechanism by which the miRNAs are included prior to leaving the cell and the possible exosomal roles are here discussed.

### 3.1. Exosome Formation

A major method for the entry of material into the cell is via endocytosis whereby a vesicular early endosome is formed with an initial internal pH of ~6.5. This can be either recycled to the plasma membrane or passaged to the Golgi apparatus or it can progress towards the nucleus into the interior of the cell when the pH of the late endosome is changed to pH 5.5 via membrane proton pumps. At this point, the vesicle can be considered as a lysosome. However, not all vesicles continue to this stage and rest with the lysosomal internal pH at 4.4–5.0. 

The control of the formation of MVBs is not completely clear. However, after becoming a late endosome, the membrane infolds to form small vesicles within, known as intra-vesicular vesicles (IVL), and the whole structure becomes an MVB. It has been suggested by Tkach and Thèry [57] that the intraluminal budding of the exosomal membrane is aided by syndecan-syntenin directly interacting with ALIX protein via the Leu-Tyr-Pro-X(n)-Leu motif. This process seems to require ubiquitination of the cytosolic tail of endocytosed receptors. Nevertheless, it appears that there are two possible pathways for the formation of exosomes. In the first, MVB formation is coordinated by ESCRT—the endosomal sorting complex required for transport and comprised of four soluble multi-protein complexes, namely ESCRT-0, ESCRT-I, ESCRT-II and ESCRT-III. Normally, they are associated with the cytosolic side of the endosomal membrane in order to sort particular proteins to the ILVs [58]. The second concerns the formation of an MBV in the absence of an ESCRT type [8]. Various studies indicate that subpopulations of MVBs’ alternative biogenesis mechanisms are dependent upon the different cell types present or, alternatively, the variety of mechanisms could all be present in the same cell type [8].

### 3.2. miRNA Entry into Forming Exosomes

Clearly, whilst miRNAs are needed for exosomal cell signaling, many miRNAs remain in the cell. Those passing into exosomes do so via a loading process that has not been completely identified. 

Argonaute proteins have been shown to be important for miRNA function as well as being a carrier of miRNAs and having an involvement in exosomal loading. However, some exosomal miRNAs have been shown to be Ago2-free among >90% of Ago protein–bound miRNAs remaining in the cell [59,60]. Exosomal miRNAs can also be completely free from miRISC. Instead, they are identified by specific proteins, e.g., hnRNPA2B1 and hnRNPA1, that recognize the specific miRNA-binding motifs so resulting in the miRNAs being selectively loaded into exosomes [61].

There are at least three potential mechanisms for the sorting of miRNAs into exosomes, although the underlying processes are not completely established. The first involves a pathway identified by Villarroya-Beltri et al. [61] in which specific miRNAs are packed into exosomes via sumoylated hnRNPA2B1 that was able to recognize the GGAG motif present in the 3′ end region of the miRNA sequences. In addition, miRNA sorting may involve two other hnRNP family proteins, hnRNPA1 and hnRNPC that can also bind to exosomal miRNAs so implicating them in the sorting process. The second approach involves the neural sphingomyelinase 2 (nSMase2)-dependent pathway. Kosaka et al. [62] found increased exosomal miRNA numbers on nSMase2 overexpression. Conversely, a reduced number of exosomal miRNAs occurred on nSMase2 expression inhibition. Similarly, two other hnRNP protein families that might be candidates to enable miRNA sorting are hnRNPA1 and hnRNPC that also bind to exosomal miRNAs. A third approach involves the findings by Koppers-Lalic et al. [63] that the 3′ ends of uridylated endogenous miRNAs were mainly present in exosomes isolated from either B cells or urine. This implies that there is possibly a critical sorting signal associated with the 3′ end of the miRNA sequence [61]. Janas et al. [64] have suggested that miRNAs are transported by RNA-binding proteins to be bound to the lipid raft-like region of the cytoplasmic leaflet of the MVB limiting membrane where miRNAs with the highest affinity to the raft-like region are retained. This could well depend upon particular binding motifs, such as the GGAG motif described by Villarroya-Beltri et al. [61], and miRNA hydrophobic modifications. When the miRNA have become attached, a spontaneous, inwards budding process from the raft-like region will occur to produce ILVs and hence, exosomes. The budding process could need the presence of ceramide molecules present in the membrane’s cytoplasmic leaflet and both lysophospholipid and glycosphingolipid molecules in the luminal leaflet [64]. 

### 3.3. Exosomal Release and Uptake by Other Cells

#### 3.3.1. Release

The precise mechanism for the release of exosomes from the MVBs at the plasma membrane has not yet been established. MVBs can be directed either to the plasma membrane for exosomal release or to lysosomes for degradation. The mechanism by which this selection process occurs is not clear. However, Villarroya-Beltri et al. [65] showed that IFN-I inhibits exosome secretion by inducing protein ISGylation of the MVB protein TSG101 that is both aggregated and degraded. In particular, IFN-I was found to induce protein ISGylation so inhibiting exosome secretion. These authors suggested that MVB protein ISGylation could result in the fusion of MVBs and lysosomes so directing MVBs to degradation rather than secretion [65]. The transport of MVBs to the plasma membrane depends upon an association with both actin and microtubule cytoskeleton elements [66,67,68]. However, Hessvik & Llorente [69] have indicated that many components are involved including several Rab GTPases and small GTPases, such as the Rho/Rac/cdc42 family, as well as proteins including SNARE proteins. Ca^2+^ has also been shown to stimulate exosome release from tumor cells [70,71]. On release, it is not necessary that exosomes travel long distances from the parent cell since they have been shown to remain clustered and attached to the plasma membrane by tetherin [72]. 

#### 3.3.2. Uptake

Currently, there are a few data regarding the uptake of exosomes by either healthy or pathological state cells e.g., cancer cells, and the mechanisms by which a cellular response is elicited.

McKelvey et al. [73] indicated three possible pathways for the uptake of miRNAs from exosomes namely, membrane fusion and release of the miRNAs, macro-pinocytosis and receptor/raft-mediated endocytosis. Of these methods, the last two have offered some possibilities. Thus, exosomes released from cultured rat adrenal gland medulla (PC12) tumors were partially internalized by clathrin-mediated endocytosis. This was shown by the use of pharmacological reduction (CPZ) and siRNA knockdown of clathrin [74]. The internalization of glioblastoma-derived exosomes occurred by lipid raft-dependent endocytosis and needed *ERK1/2-HSP27* signaling [75]. However, when the endocytic pathway(s) required for the exosome uptake were studied with different pharmacological inhibitors of caveolin-dependent, clathrin-dependent and macro-pinocytosis pathways, no such pathways appeared to be significantly involved in the internalization of exosome-associated alpha-synuclein oligomers [76].

### 3.4. Exosomal Functions in Healthy and Cancer Cells

Hessvik and Llorente [69] have discussed the role of cell stress as a stimulant for the increased release of exosomes occurring after either cisplatin treatment or irradiation. The increased volume of exosomes released may be due to the need to remove “waste” from the stressed cells or be an indication of signaling the stress situation to adjacent cells. 

Exosomes can be released from a broad range of cell types and appear to circulate to both local and distant cells where they can act in different roles, e.g., in the immune response, tumor progression and neurodegenerative diseases. Thus, studies on the nervous system [77] have indicated that they may be implicated as a novel form of intercellular communication within the nervous system where both glial cells and neurons secrete exosomes under glutamate regulation. Their release has been shown to occur either pre-synaptically at the neuromuscular junction [78,79] or post-synaptically by cortical neurons upon activation of synaptic N-methyl-D-aspartic acid (NMDA) receptors, and then binding pre-synaptically to hippocampal neurons [80]. They may also be exploited as biomarkers in central nervous system diseases [77].

The wide variety of studies on the miRNAs loaded into exosomes by different cell types – both healthy and cancer – all demonstrate that a sorting mechanism guiding specific intracellular miRNAs to enter exosomes is present in the parent cells [81]. Thus, different levels of eight exosomal miRNAs, including miR-21 and miR141, were seen between benign tumors and ovarian cancers [82].

However, the functions of exosomal miRNAs, in general, can be considered in two ways. The first, when miRNAs perform the conventional negative regulation to confer changes in the expression levels of target genes, e.g., reduced *ZO-1* gene expression in endothelial cells and the promotion of lung and brain metastases by exosomal miR-105 released from the breast cancer cell lines MCF-10A and MDA-MB-231 [83]. Alternatively, miRNAs may execute different actions when they are considered as exosomal miRNAs rather than intracellular miRNAs. Thus, exosomal miR-21 and miR-29a, in addition to targeting mRNA, were shown to act as ligands binding to toll-like receptors (TLRs) and activating immune cells—a completely different role for miRNAs [84]. 

In the case of miRNAs and cancer, there are three primary involvements possible: tumor suppression, tumor initiation and tumor cell preparation. Thus, miR-146 appears to acts as a tumor suppressor in prostate, glioma, breast and pancreatic cancers [85,86,87,88] with its overexpression in e.g., stromal cells triggering its exosomal release [89]. In contrast, exosome function is also involved in the intercellular transmission of oncogenically acting miRNAs. Thus, in many cancer cells, oncogenic miR-21 is exosomally secreted. This is one example in a range of tumor cells [90,91] in which its expression is upregulated during tumor progression and metastasis [92,93]. Hence, it is possible that the tumor-inducing miRNAs can either initiate tumors in distant cells or merely initiate a pre-tumor state in such cells.

### 3.5. Exosomal miRNAs in Cell-to-Cell Communication 

For the first time, in 2007, the occurrence of miRNAs in exosomes was described by Valadi et al. who called them “exosomal shuttle miRNAs”. They observed that exosomes contained multiple and heterogeneous RNA species including miRNAs in exosomes from mouse mast cells. Among these species, they detected a panel of approximately 121 miRNAs, including let-7, miR-1, miR-15, miR-16, miR-181 and miR-375. Some of these exosomal miRNAs were enriched at higher levels in exosomes than in the mast cells, implying that miRNAs may be exclusively packed into exosomes [94]. The study by Valadi et al. paved the way for numerous further in vitro as well as in vivo investigations that provided evidence on the spreading of miRNAs from donor cells to adjacent cells in the tumor microenvironment via exosomes and their influence on tumor progression and metastasis [95]. The following survey summarizes these exciting findings on cell-to-cell communication with an emphasis on breast, ovarian and prostate cancer cells (Table 1).

#### 3.5.1. Breast Cancer (BC)

Despite improved therapeutic strategies and progress in cancer medicine in the last years, BC-associated death ranks as the second highest cause of death in women, metastasis accounting for 90% of BC mortality and resistance to treatment. In this heterogeneous disease comprising over 90% of ductal and lobular BC subtypes, various cellular pathways are dysregulated leading to diverse causes of the disease. In order to assign an adequate therapy to the BC patient, immunohistochemistry tests for estrogen receptor (ER), progesterone receptors (PR), human epidermal growth factor receptor 2 (HER2) and Ki-67 protein expression are applied to classify BC into different groups [96]. Systemic therapies based on a hormone or cytotoxic agents and target antibodies are initially effective in controlling tumor progression, but they then generate resistance to treatment [97]. In this regard, growing evidence points to a role for exosomes and miRNAs in BC progression, metastasis and resistance.

In the tumor microenvironment, multiple juxtacrine and paracrine interactions occur between cancer and non-cancer cells impacting both BC metastasis and response to therapy [98]. The major integral components of the BC stroma are cancer-associated fibroblasts (CAFs) that drive tumor progression. For the first time, Donnarumma et al. provided evidence of the function of CAF exosomes and their miRNAs in the induction of the stemness and epithelial-mesenchymal transition (EMT) phenotype in different BC cell lines. They performed differential expression profile analysis and identified three upregulated miRNAs (miR-21, miR-378e and miR-143) in CAF exosomes as compared with *wt* fibroblast exosomes. Immunofluorescence studies indicated that exosomes were transferred from CAFs to BT549, MDA-MB-231 and T47D cells. The release of their miRNA cargo into these BC cell lines affected the character of the recipient cells that exhibited a significantly increased capacity to form mammospheres, stem cells and EMT markers, plus anchorage-independent cell growth. These effects could be reversed by transfection with the corresponding anti-miRNAs. In line with these observations, normal fibroblast exosomes transfected with miR-21, miR-378e and miR-143 promoted the stemness and EMT phenotype of BC cells [99]. Furthermore, Baroni et al. showed that miR-9, upregulated in various BC cell lines and identified as a pro-metastatic miRNA, affected the properties of human breast fibroblasts, enhancing the switch to a CAF phenotype, so contributing to tumor growth. Tumor-secreted miR-9, which is also secreted by fibroblasts, was transferred via exosomes to recipient *wt* fibroblasts. The uptake resulted in increased fibroblast motility, possibly by inhibiting its direct target, the adhesion molecule E-cadherin the loss of which is involved in EMT [100]. Shah et al. performed experiments in ER-positive MCF-7 cells and treated them with conditioned medium from CAFs derived from ER-negative primary breast tumors. This treatment provoked ER-positive MCF-7 cells to repress ER. Nanoparticle tracking analysis and transmission electron microscopy (TEM) confirmed the presence of CAF-secreted exosomes in the conditioned medium and the uptake of these exosomes by the ER-positive MCF-7 cells. Collectively, they demonstrated that CAF-secreted exosomal miRNAs, miR-221 and miR-222, are directly involved in ER-repression and contribute to the MAPK (mitogen-activated protein kinase)-induced ER repression in BC cells [101].

Up-regulation of miR-210 induced by a hypoxic microenvironment was recently reported to promote BC stem cells metastasis, proliferation and self-renewal by targeting E-cadherin [102]. Jung et al. used a miR-210 specific reporter system and a fluorescent dye to visualize exosome-mediated transfer of miR-210 from hypoxic BC to neighboring cells. They observed that exosomes containing miR-210 were transferred to cells in the tumor microenvironment and that miR-210 was involved in the expression of vascular remodeling related genes, such as Ephrin A3 and protein tyrosine phosphatase PTP1B, to promote angiogenesis in tumor-bearing mice [103]. Applying scanning electron and confocal microscopy, Singh et al. demonstrated that either nSMase2 or ceramide involved in exosome generation promoted exosome-mediated miR-10b secretion, whereas its ceramide inhibitor suppressed this secretion. The uptake of miR-10b, which plays an important role in metastasis, suppressed the protein level of its target genes, such as the homeobox gene *HOXD10* and *KLF4* (Krüppel-like factor 4), in recipient cells. Additionally, the treatment of non-malignant HMLE cells with exosomes derived from the highly aggressive, invasive and poorly differentiated triple-negative BC cell line MDA-MB-231 could induce the invasiveness of HMLE cells [104]. Furthermore, Zhou et al. detected that the transfer of exosomal miR-105 to non-metastatic BC cells induced metastasis and vascular permeability by targeting the cellular tight junction protein *ZO-1* [83]. Yang et al. found that in co-cultured BC cells, IL-4 (interleukin-4)-activated macrophage miR-233 could increase the invasive capacity of recipient cells [105]. O’Brien et al. investigated the impact of exosomal miR-134 on the signal transducers and activators of transcription 1B (*STAT1B*), which is a key component of the JAK/STAT pathway, and is one of the transcription factors that regulate the chaperone protein Hsp90α [106]. Evidently, the delivery via miR-134-enriched exosomes inhibited *STAT5B* and *Hsp90* which, in turn, reduced cellular migration and invasion and enhanced sensitivity to anti-Hsp90 drugs in recipient cells [107].

Drug resistance is a major obstacle to successful cancer treatment. To date, a number of studies have shown that exosomes act as mediators of drug resistance by transferring specific miRNAs into sensitive recipient cells. Following binding, absorption and internalization, exosomes may change chemo-susceptibility in recipient sensitive cells by modulating gene expression, signaling pathways, cell cycle distribution and drug-induced apoptosis [108]. The oncomiRs miR-221 and miR-222 are involved in resistance to tamoxifen [109]. Tamoxifen, which binds to and inhibits the ER, has been the first targeted drug and the gold standard in the treatment of ER-positive BC for almost 50 years [110]. Wei et al. applied TEM and nanoparticle tracking and detected that there were significant differences in the concentration and size distribution of exosomes derived from tamoxifen-resistant MCF-7 cells compared with those from sensitive MCF-7 cells. Fluorescently-labeled exosomes from resistant MCF-7 cells could enter *wt* MCF-7 cells. Subsequently, the elevated miR-221/222 levels in the recipient cells reduced the target gene expression of the cyclin-dependent kinase inhibitor *p27* and *ERα*, leading to deregulation of the cell cycle and resistance to tamoxifen [111]. To date, the laboratory of Tang has performed several studies that examined the ability of drug-resistant BC cells to transmit the resistance capacity of cytostatic docetaxel and anthracycline adriamycin to sensitive cells by exosomes [112]. In vitro studies showed that possibly exosomal miR-100, miR-222 and miR-30a are involved in the exosome shuttle to transfer chemoresistance from cell to cell [113]. Using microarray and PCR, these scientists [114] found that exosomes from docetaxel-resistant BC cells spread chemoresistance to fluorescent sensitive cells. Target gene prediction and pathway analysis revealed the involvement of twenty most abundant miRNAs in exosomes derived from docetaxel-resistant BC cells in pathways implicated in therapy failure. These miRNA-containing exosomes were able to downregulate mRNAs in fluorescent sensitive cells, such as the inhibitors of the Ras/MAPK pathway *Sprouty2* and *PTEN* (phosphatase and tensin homologue), known to be targeted by miR-23a [115], miR-222 [116], and miR-452 [117]. The same laboratory detected that miRNA-containing exosomes derived from adriamycin-resistant BC cells were also able to increase the overall resistance of fluorescent, sensitive cells to adriamycin exposure. The levels of miRNAs selectively packaged into exosomes from adriamycin-resistant BC cells were significantly increased in recipient fluorescent, sensitive cells [118]. On the other hand, drug resistance can be reversed by the sesquiterpene β-elemene. In this regard, the laboratory of Tang showed that β-elemene mediated increased and decreased expression of MDR (multidrug resistance)-specific miR-34a and miR-452, respectively, resulting in increased expression of *PTEN* and decreased expression of P-glycoprotein in both cells and exosomes. β-elemene effectively sensitized drug-resistant BC cells to docetaxel and adriamycin so reducing chemoresistance transmission via exosomes to other BC cells, and induced a significant increase in the apoptotic rate [119]. Furthermore, exosomes may also contribute to the antitumor response during BC neoadjuvant chemotherapy via miRNA transfer. Bovy et al. revealed the involvement of the endothelium in the modulation of tumor development via the secretion of exosomal miR-503 in response to chemotherapy [120].

DHA (docosahexaenoic acid) is a natural compound with anticancer and anti-angiogenesis activity [121]. Hannafon et al. observed that treatment of MCF7 and MDA-MB-231 cells with DHA caused an increase in exosome secretion and exosomal miRNAs. The most abundant exosomal miRNAs were let-7a, miR-23b, miR-27a/b, miR-21, let-7 and miR-320b. When DHA-treated MCF7 cells were co-cultured with or their exosomes were directly applied to endothelial cell cultures, an increase in the expression of these miRNAs was observed in the endothelial cells. In addition, overexpression of miR-23b and miR-320b in endothelial cells decreased the expression of their pro-angiogenic target genes, such as *PLAU* (plasminogen activator, urokinase), *AMOTL1* (angiomotin), *NRP*1 (neuropilin) and *ETS2*, and significantly inhibited tube formation by endothelial cells so suggesting that these miRNAs transferred by exosomes mediated anti-angiogenic action by DHA. These effects could be reversed by knockdown of the Rab GTPase *Rab27A* which controls exosome release [122]. DRβ-H (D-Rhamnose β-hederin) is an oleanane-type triterpenoid saponin isolated from the traditional Chinese medicinal plant *Clematis ganpiniana*. Chen et al. observed that DRβ-H exhibited anti-proliferative and pro-apoptotic activity in BC cells. DRβ-H was able to inhibit the secretion of exosomes containing miR-130a and miR-425, the levels of exosomes being positively associated with cell growth after absorption and internalization by target BC cells [123]. EGCG (catechin epigallocatechin gallate) contained in green tea has anti-tumor and antimicrobial properties. Jang et al. [124] showed that EGCG up-regulated miR-16 in 4T1 BC cells and their exosomes. In a murine BC model, EGCG suppressed tumor growth which was associated with decreased M2 macrophage infiltration and polarization. Ex vivo incubation of tumor-associated macrophages isolated from murine tumor graft with exosomes from EGCG-treated 4T1 cells inhibited the activation of the NF-κB pathway [125] and modulated the levels of cytokines. Treatment of tumor cells or tumor-associated macrophages with exosomes derived from EGCG-treated and miR-16-knock-down 4T1 cells restored the effects [124].

Mesenchymal stem cells (MSC) potentially differentiate into multiple cell types and are involved in formation and modulation of the tumor stroma [126]. Pakravan et al. characterized exosomes derived from MSC by scanning electron microscopy, dynamic light scattering and Western blotting. They found that miR-100 was enriched in MSC-derived exosomes and that its transfer to BC cells was associated with the down-regulation of *VEGF* in a time-dependent manner. The putative role of exosomal miR-100 transfer in regulating *VEGF* expression was substantiated by the ability of anti-miR-100 to rescue the inhibitory effect of MSC-derived exosomes on the expression of *VEGF* in BC cells [127]. In vitro and in vivo, Lee et al. demonstrated that MSC-derived exosomes enriched with miR-16 also down-regulated the expression of *VEGF* in BC cells, leading to suppression of angiogenesis. miR-16, a miRNA known to target *VEGF*, was partially responsible for the anti-angiogenic effect of MSC-derived exosomes [128].

Many years after resection of the primary tumor, BC patients can develop metastatic disease since the proliferation of disseminated tumor cells is slowed. These dormant cells are often undetectable and unresponsive to traditional chemotherapies [129]. Ono et al. suggested that exosomal transfer of miRNAs from bone marrow (BM) may promote BC cell dormancy in a metastatic niche. They established a BM-metastatic human BC cell line (BM2) and co-cultured it with BM-MSC, BM-MSC-conditioned medium and exosomes isolated from BM-MSC. All 3 constituents suppressed the proliferation of BM2 cells, decreased the abundance of stem cell-like surface markers, inhibited their invasion and decreased their sensitivity to docetaxel. The acquisition of this dormant phenotype in BM2 cells occurred by exosomes from BM-MSC cultures which were taken up by BM2 cells. Overexpression of miR-23b, the levels of which were increased in these exosomes, induced the dormant phenotype in BM2 cells through the suppression of its target mRNA, *MARCKS* (myristoylated alanine-rich C-kinase substrate), which is a major substrate of protein kinase C and encodes a protein that promotes cell cycling and motility [130]. Bliss et al. reported that BC cells prime MSC to release exosomes containing distinct miRNAs, such as miR-222 and 223, which in turn promote dormancy in a subset of BC cells and confer drug resistance. To target dormant BC cells, they developed a nontoxic therapeutic strategy based on systemic administration of MSC loaded with antagomiR-222/223. In an immunodeficient mouse model of dormant BC cells, the intravenous injection sensitized BC cells to carboplatin-based therapy and increased host survival [131].

In summary, these findings on the exosome shuttle indicate that miRNAs may be used as an anticancer therapeutic tool, and exosomes may serve as delivery systems for clinical application. In this regard, Ohno et al. [132] showed that exosomes efficiently delivered antitumor miRNAs to BC tissues in vivo. Since human tumors of epithelial origin often display elevated *EGFR* (epidermal growth factor receptor) expression, they used *EGFR* as a receptor target in the cancer drug delivery system and GE11 peptide as an alternative to the ligand EGF with its strongly mitogenic and neoangiogenic character [133]. The scientists transfected donor cells with let-7a, purified the exosomes from the culture supernatant and modified their surface with the peptide to deliver the miRNA to EGFR-expressing cancer tissues. Intravenously injected and fluorescently labeled exosomes delivered let-7a specifically to xenograft BC cells in a mouse model, suggesting that exosomes can be used therapeutically to target EGFR-expressing cancerous tissues with nucleic acid drugs [132].

#### 3.5.2. Epithelial Ovarian Cancer (EOC)

In the female reproductive system, EOC is one of the most aggressive carcinomas and the leading cause of death among gynecologic malignancies due to its frequent recurrence. More than two-thirds of patients are diagnosed at advanced tumor stages and have a 5-year survival rate of less than 40% [134]. Current diagnostic methods for detection and monitoring of EOC mainly include pelvic examination, transvaginal ultrasound and measurement of the serum biomarker CA125 (carbohydrate antigen 125) [135]. Standard treatment for advanced stage EOC is tumor-debulking surgery and adjuvant platinum-based chemotherapy. However, after complete surgery and chemotherapy, the risk of relapse is approximately 85% [136].

Apart from BC-derived exosomes, EOC-derived exosomes also deliver miRNAs to induce M2 macrophage polarization under hypoxic conditions. Based on microarray analysis, Chen et al. showed the enrichment of miR-21, miR-125b and miR-181d in hypoxic exosomes. These three highly expressed miRNAs were induced by hypoxia via the hypoxia-inducible factor (HIF), promoting EOC cell proliferation and migration. [137]. In addition, Ying et al. also observed that EOC-derived exosomes activated macrophages to a tumor-associated macrophage-like phenotype. Exosomes enriched with miR-222 and released from EOC cells were transferred to macrophages. In the recipient macrophages, overexpression of miR-222 induced polarization of the M2 phenotype. Down-regulation of *SOCS3* (suppressor of cytokine signaling 3) mediated by miR-222 correlated with an increased *JAK* (Janus kinase)-*STAT3* (signal transducers and activators of transcription 3) activation which could facilitate the progression of EOC [138]. Hu et al. stimulated macrophages with TWEAK (tumor necrosis factor-related weak inducer of apoptosis), a ligand of the TNF receptor superfamily. They detected that exosomes from TWEAK-stimulated macrophages were internalized by EOC cells and inhibited cell metastasis, as well as blocking EOC metastasis in a xenograft mouse model. TWEAK-stimulated macrophages inhibited metastasis via shuttling exosomal miR-7 to EOC cells, thereby inhibiting the EGFR/AKT/ERK1/2 pathway [139]. This laboratory also found that miR-7 was able to reverse EMT through AKT/ERK1/2 pathway inactivation by inhibiting *EGFR* expression in EOC cell lines [140].

Performing a study on MSC, Reza et al. showed that exosomes from conditioned medium of human adipose MSC exhibited inhibitory effects on EOC cells by blocking the cell cycle, and activating mitochondria-mediated apoptosis signaling. MSC-derived exosomes induced apoptosis signaling by upregulating different pro-apoptotic signaling molecules, such as the *BCL2* inhibitor *BAX*, and the caspases *CASP9* and *CASP3*, as well as by downregulating the anti-apoptotic protein *BCL2*. Using next-generation sequencing (NGS), they detected a rich population of exosomal miRNAs, with the most abundantly expressed miRNAs being miR-4792, miR-320b and miR-320a [141]. Au Yeung et al. performed experiments on adipocytes and fibroblasts and demonstrated that the malignant phenotype of metastatic EOC cells was altered by miR-21 delivered by exosomes from neighboring stromal cells in the omental tumor microenvironment. Functional studies revealed that miR-21 was transferred via exosomes from cancer-associated adipocytes and CAFs to the cancer cells, suppressed ovarian cancer apoptosis and conferred chemoresistance by binding to its direct target *APAF1* (apoptotic protease-activating factor 1) [142].

Amniotic fluid stem cells have been reported to have the potential to treat chemotherapy-induced premature ovarian failure in women [143]. Xiao et al. found that exosomes derived from amniotic fluid stem cells prevented ovarian follicular atresia in mice treated with chemotherapy via the delivery of miRNAs of which miR-146a and miR-10a were highly enriched. The down-regulation of both miRNAs in exosomes from amniotic fluid stem cells attenuated the anti-apoptotic effect on granulosa cells damaged by chemotherapy in vitro [144]. Vaksman et al. observed an enlarged mesothelial clearance area which may simulate modulation of tumor cell invasion in the peritoneum of EOC patients following exposure of the mesothelial cell layer to exosomes from effusion fluid. This process was mediated by miR-21 and miR-29a. In an immunodeficient mouse model, these exosomes affected both tumor cells and cells in the tumor microenvironment and induced more aggressive, infiltrative tumors [145].

#### 3.5.3. Prostate Cancer (PCa)

In industrialized countries, PCa is the most common malignancy in men. The current gold standard for diagnosis and response to treatment is still PSA (prostate-specific antigen) screening. However, the PSA test has limitations, because elevated serum PSA values are associated with the prostate volume. Thus, they do not differ between malignant and benign prostate tumors. The majority of PCa patients is diagnosed at the age above 65 and display localized (within the prostate) or regionally confined (spread into immediate surrounding tissue) tumors [146]. Curative therapies for PCa patients at early tumor stages are surgery and radiation, but approximately one-third of the patients develop a biochemically recurrent disease (rising PSA levels) which is treated with androgen deprivation therapy (ADT) [147]. However, after an initial response, PCa patients eventually become refractory to ADT. These castration-resistant PCa patients are treated with androgen blockade and/or chemotherapy which allow each second patient to survive. However, the other patients are most likely to form metastasis [148].

To understand the development of PCa, Kosaka et al. performed in vitro and in vivo experiments and documented that tumor-suppressive miRNAs can be implicated in cell competition between cancer cells and non-cancer cells. They transferred exosomal miR-143 from a non-cancerous prostate cell to a PCa cell and showed that this miRNA acted as a tumor suppressor by repressing the growth in recipient PCa cells [149].

Ye et al. demonstrated that exosomal miR-141-3p promoted osteoblast activity as mice injected with miR-141-3p-enriched exosomes developed apparent osteoblastic bone metastasis. Using confocal imaging, they observed that exosomes from MDA PCa 2b cells entered osteoblasts, and miR-141-3p was transferred to osteoblasts through these exosomes in vitro. Exosomal miR-141-3p from MDA PCa 2b cells promoted osteoblast activity and increased the expression of *osteoprotegerin*, a soluble decoy receptor for TRAIL (TNF-related apoptosis-inducing ligand). In addition, miR-141-3p suppressed the protein levels of its target gene *DLC-1* (deleted in liver cancer 1), a member of the RhoGTPase-activating proteins, indicating its relevance in activating the p38MAPK pathway [150].

In metastatic PCa, the gene encoding the cytoskeletal regulator *DIAPH1* (diaphanous homolog 1), a regulator of cytoskeletal dynamics [151], is lost at high frequency. Kim et al. observed that stimulation of LNCaP cells with the prostate stroma-derived growth factor HB-EGF (heparin-binding EGF-like growth factor) combined with p38MAPK inhibition caused exosome release, a process mediated by ERK1/2 hyperactivation. Increased exosome release mediated by DIAPH3 silencing in DU145 cells resulted in activation of AKT1 and androgen signaling, increased proliferation of recipient tumor cells and suppressed proliferation of human macrophages and peripheral blood mononuclear cells. DU145-derived exosomes contained miR-125a that suppressed *AKT1* expression and proliferation in recipient human peripheral blood mononuclear cells and macrophages [152].

## 4. Conclusions

In cancer, exosomes with their cargo of miRNAs are released from different sources, amongst others from heterogeneous areas of the primary tumor, metastases or other organs affected by tumor burden. Exosomal miRNAs have multiple functions leading to tumor cell development, growth, migration, invasion, dissemination and metastasis, impairment of the immune system response and/or drug resistance [95,108,153]. Their signatures reflect miRNA expression profiles of tumor cells of origin [82], but they also point to a selective packaging of miRNAs into exosomes as a mechanism to coordinate activation of tumor progression and metastatic cascade [154]. The central role of exosomes in tumor progression has been highlighted by the fact that breast cancer exosomes can perform cell-independent miRNA biogenesis and so stimulate non-tumorigenic epithelial cells to form tumors by altering their transcriptome in a Dicer-dependent manner [155]. In particular, the ability of exosomes to transfer miRNAs from cell to cell, to modulate the characteristics of the recipient cells and induce malignant transformation makes exosomes and miRNAs attractive molecular biomarkers in cancer diagnosis and prognosis [95]. Repeated blood collections display changes in the cargo of miRNAs in exosomes and also make them interesting as candidates for therapeutic target molecules for clinical application [9]. Downregulation of target oncogenes by re-expression of tumor suppressor miRNAs, or re-expression of tumor suppressor genes by silencing of oncogenic miRNAs, has been anticipated to impair tumor progression and metastasis. Restoring and blocking miRNA function may be performed by replacement of tumor suppressor miRNAs with either synthetic or viral vectors encoded for miRNA mimics or by antisense-mediated inhibition of oncogenic miRNAs, respectively. Moreover, a recent study has summarized the potential use of exosomes as a biological vehicle system for the delivery of various drugs, such as doxorubicin, siRNAs, and miRNAs [156]. To avoid cancer propagation, a further therapeutic application may be blocking of the secretion of tumor cell-derived exosomes. Regardless of the interest that has been generated in the research field of exosome shuttles, there are still gaps in our knowledge. Therefore, controls and numbers of exosome release and uptake dynamics, as well as the fates of those exosomes in the microenvironment are still not well-known. In addition, the mechanism by which exosomes are directed to particular cells in vivo still has to be clarified. It should also be kept in mind that exosomal miRNAs have numerous biological functions in both their cells of origin and the recipient cells. A single miRNA can target numerous mRNAs and, consequently, prevent the translation of many different proteins that are involved in several cancer-relevant signal transduction pathways. Finally, the interplay of exosomal miRNAs with their different functions requires further extensive research.

Thus, the understanding of the biology of the exosome shuttle with its cargo of miRNAs may be an essential step towards an improved cancer diagnostics and personalized therapy. However, there is still a long way to go to achieve the goal of developing exosome- and miRNA-based cancer biomarkers and therapeutic strategies.

## Figures and Tables

**Table 1:** Exosome shuttle from cell to cell.

Donor Cells	Recipient Cells	miRNAs	mRNA Targets	Functions	Ref.
**Breast Cancer**					
CAFs	BT549, MDA-MB-231, T47D	miR-21, miR-378e, miR-143	n.d.	formation of mammospheres, increase in stem cell and EMT markers, anchorage-independent cell growth	[99]
CAFs	wt fibroblasts	miR-9	E-cadherin	increase in motility	[100]
CAFs	MCF-7	miR-221, miR-222	ER	repression of ER	[101]
hypoxic MCF-7	stem cells	miR-210	E-cadherin	induction of metastasis, proliferation and self-renewal	[102]
hypoxic 4T1	BALB/c mice	miR-210	Ephrin A3, PTP1B	changes in vascular structure to promote angiogenesis	[103]
MDA-MB-231	non-malignant HMLE	miR-10b	HOXD10, KLF4	induction of invasion	[104]
MDA-MB-231	MCF-10A	miR-105	ZO1	induction of metastasis and vascular permeability	[83]
HEK-293T	SKBR3, MDA-MB-231	miR-223	Mef2c-β-catenin pathway	increase in invasion	[105]
Hs578T	Hs578Ts(i)_8_	miR-134	STAT5B, Hsp90	reduction of migration, invasion	[107]
resistant MCF-7	wt MCF-7	miR-221, miR-222	p27, ERα	resistance to tamoxifen, deregulation of the cell cycle	[111]
resistant MCF-7	wt MCF-7	miR-100, miR-222, miR-30a	PTEN	resistance to adriamycin and docetaxel, modulation of cell cycle distribution and drug-induced apoptosis	[113]
resistant MCF-7	wt MCF-7	miR-1246, miR-23a, miR-1469, miR-638, miR-1915, miR-2861, let-7a/b, miR-24, miR-149*, miR-3178, miR-3196, miR-16, miR-23b, miR-762, miR-663, let-7c, miR-26a, miR-27a, miR-1908	MAPK, Wnt, TGF-ß pathways	resistance to docetaxel	[114]
DHA-treated MCF-7	endothelial EA.hy926	let-7a, miR-23b, miR-27a/b, miR-21, let-7, and miR-320b	PLAU, AMOTL1, NRP1 and ETS2	transfer of DHA’s anti-angiogenic action, inhibition of tube formation	[122]
MSC	BALB/c mice	miR-16	VEGF	Inhibition of angiogenesis	[128]
BM-MSC	BM2	miR-23b	MARCKS	suppression of proliferation, decrease in stem cell-like surface markers and sensitivity to docetaxel, inhibition of invasion	[130]
HEK293	RAG2^–/–^ mice	let-7a	HMGA2	inhibition of tumor development	[132]
**Ovarian Cancer**					
hypoxic SKOV3	nude mouse	miR-21–3p, miR-125 b-5p, miR-181 d-5p	SOCS4/5/STAT3 pathway	induction of proliferation and migration	[137]
SKOV3	athymic nude mice	miR-222	SOCS3	cancer progression	[138]
TWEAK-stimulated THP-1	athymic nude mice	miR-7	EGFR/AKT/ERK1/2 pathway	inhibition of metastasis	[139]
CAFs and CAAs	SKOV3	miR-21	APAF1	suppression of apoptosis, resistance to paclitaxel	[142]
Amniotic fluid stem cells	Chemotherapy treated mice	miR-10a, miR-146	n.d.	inhibition of apoptosis and ovarian follicles from atresia	[144]
Cancer effusion	immunodeficiency mice	miR-21, miR-29a	n.d.	induction of aggressive, infiltrative tumors	[145]
**Prostate Cancer**					
PNT-2	PC-3M-luc cells	miR-143	n.d.	repression of growth	[149]
MDA PCa 2b	BALB/C mice	miR-141-3p	DLC-1	osteoblast activity, increase in osteoprotegerin	[150]

The Table lists the studies analyzing the exosome shuttle by co-culture experiments in vitro or in mice. AMOTL1, angiomotin L1; APAF1, apoptotic protease-activating factor 1; BM2, bone marrow-metastatic human BC cell line; CAAs, cancer-associated adipocytes; CAFs, cancer-associated fibroblasts; DHA, docosahexaenoic acid; DLC-1, deleted in liver cancer 1; EGFR, epithelial growth factor receptor; EMT, epithelial-mesenchymal transition; ERK1, extracellular signal-regulated kinase 1; ER, estrogen receptor; HMGA2, high mobility group AT-hook 2; HOXD10, homeobox D10; Hsp90 heat shock protein 90; KLF4, Krüppel-like factor 4; MAPK, mitogen-activated protein kinase; MARCKS, myristoylated alanine-rich C-kinase substrate; MSC, mesenchymal stem cells; NRP1, neuropilin 1; PLAU, plasminogen activator, urokinase; PTEN, phosphatase and tensin homologue; PTP1B, protein tyrosine phosphatase 1B; SOCS3, suppressor of cytokine signaling 3; STAT5B, signal transducers and activators of transcription 5B; TGF-ß transforming growth factor ß; VEGF, vascular endothelial growth factor; n.d. not determined.
